# LINC01871 facilitates cervical cancer cell migration and immune escape by targeting miR‐873‐3p/MAP3K2 axis

**DOI:** 10.1002/kjm2.12948

**Published:** 2025-03-04

**Authors:** Yan Li, Li Wei, Hui‐Hui Zhang, Hong‐Fei Ci, Duo‐Jie Li

**Affiliations:** ^1^ Department of Oncology Gynecology The First Affiliated Hospital of Bengbu Medical University Bengbu China; ^2^ Department of Pathology The First Affiliated Hospital of Bengbu Medical University Bengbu China; ^3^ Department of Radiotherapy The First Affiliated Hospital of Bengbu Medical University Bengbu China

**Keywords:** cervical cancer, immunity, invasion, LINC01871, MAP3K2

## Abstract

Cervical cancer (CC) poses a significant threat to women's health and lives worldwide. Emerging evidence indicates that long noncoding RNA LINC01871 is closely associated with immune regulation in CC patients. However, its specific role and underlying mechanisms in CC remain poorly understood. In this study, we examined the expression levels and subcellular localization of LINC01871 in CC cell lines. Functional assays, including transwell migration and invasion assays as well as macrophage phagocytosis assays, were conducted to estimate CC cell invasiveness, migration, and immune response. Western blotting was performed to assess the protein expression of markers associated with epithelial–mesenchymal transition (EMT), immunity, and MAPK signaling. A luciferase reporter assay was used to confirm the interactions between LINC01871, miR‐873‐3p, and MAP3K2. Additionally, a xenograft mouse model was employed to investigate the in vivo effects of LINC01871 on CC progression. Our results revealed that LINC01871 is highly expressed and predominantly localized in the cytoplasm of CC cell lines. LINC01871 knockdown significantly suppressed CC cell invasion, migration, EMT, and immune escape in vitro and reduced tumor growth in vivo. Mechanistically, LINC01871 was found to interact with miR‐873‐3p, leading to the upregulation of MAP3K2 and subsequent activation of MAPK signaling. Furthermore, MAP3K2 overexpression rescued the inhibitory effects of LINC01871 silencing on the malignant behaviors of CC cells. In conclusion, LINC01871 facilitates CC metastasis by driving EMT and modulating the immune response via the miR‐873‐3p/MAP3K2/MAPK signaling pathway.

## 1 INTRODUCTION

Cervical cancer (CC) is the fourth most common malignancy among women globally. In 2020, approximately 604,000 new cases of CC were diagnosed, and an estimated 342,000 deaths were attributed to this disease worldwide.^1^ Persistent infection with human papillomavirus (HPV) is the leading cause of CC, and the introduction of HPV vaccination has significantly reduced the incidence of CC.^2^ Over the past decades, substantial progress has been achieved in the development of therapeutic strategies for CC. Current treatment modalities include surgery, radiotherapy, chemotherapy, targeted therapies, and immunotherapy.^3^

Immunotherapy has emerged as a promising antitumor strategy for CC, leveraging the immune system to target and eliminate cancer cells.^4^ However, cancer cells often evade immune surveillance by expressing immune checkpoint molecules such as programmed death‐ligand 1 (PD‐L1) and cluster of differentiation 47 (CD47), thereby suppressing antitumor immune responses.^5^ In addition, epithelial–mesenchymal transition (EMT), a highly conserved biological process, plays a critical role in facilitating metastasis across various cancer types, including CC.^6^ Understanding the mechanisms driving immune evasion and EMT in CC cells is therefore crucial for developing more effective therapeutic interventions for this malignancy.

Long noncoding RNAs (lncRNAs) are transcripts longer than 200 nucleotides that do not encode proteins.^7^ These molecules regulate gene expression at various levels, including chromatin remodeling, transcription modulation, and post‐transcriptional processes.^8^ lncRNAs play critical roles in immune responses and tumorigenesis.^9^ Emerging evidence suggests that lncRNAs contribute to the establishment of an immunosuppressive microenvironment through specific mechanisms, thereby facilitating tumor evasion from immune surveillance.^10^ In various cancers, including CC, lncRNAs exhibit either oncogenic or tumor‐suppressive functions. For instance, Hu et al.^11^ demonstrated that lncRNA LINC01287 facilitates CC progression and is associated with poor prognosis in patients. Conversely, Qin et al.^12^ demonstrated that lncRNA LINC00657 significantly suppresses the aggressiveness of CC cells. Notably, several studies have identified LINC01871 as an immune‐related lncRNA and a potential independent prognostic biomarker for CC.^13^, ^14^ Nonetheless, the precise role and mechanisms of LINC01871 in CC pathogenesis remain to be elucidated.

In this study, we aim to investigate the function and molecular mechanisms of LINC01871 in CC. We hypothesize that LINC01871 may influence EMT and immune evasion in CC by modulating its downstream targets. Our findings are expected to provide novel insights into potential therapeutic strategies for CC.

## 2 MATERIALS AND METHODS

### 2.1 Clinical specimens

Thirty paired CC tissue samples and adjacent nontumor tissues were collected from the First Affiliated Hospital of Bengbu Medical University. None of the patients had received chemotherapy or radiotherapy before surgery. The tissue samples were immediately frozen in liquid nitrogen following surgery and stored at −80°C for subsequent analysis. Written informed consent was obtained from all participants, and the study was approved by the Ethics Committee of the First Affiliated Hospital of Bengbu Medical University (2022‐243).

### 2.2 Cell culture

Human CC cell lines (HeLa, CaSki, SiHa, C33A) and the normal cervical epithelial cell line (H8) were purchased from WheLab (Shanghai, China). The H8 and CaSki cell lines were cultured in RPMI‐1640 medium (M0200, WheLab), and HeLa, SiHa, and C33A cell lines were maintained in minimal essential medium (MEM; M0300, WheLab). All media were supplemented with 10% fetal bovine serum (FBS; Gibco, Grand Island, NY). Cells were cultured in a humidified incubator with 5% CO_2_ at 37°C.

### 2.3 Cell transfection

Short hairpin RNA targeting LINC01871 (sh‐LINC01871) and its corresponding negative control (sh‐NC) were used for LINC01871 knockdown experiments. miR‐873‐3p mimics (or miR‐NC) and the MAP3K2 overexpression vector (or empty vector) were employed to overexpress miR‐873‐3p and MAP3K2, respectively. All plasmids were purchased from RiboBio (Guangzhou, China). HeLa and CaSki cells were transfected with these plasmids using Lipofectamine 3000 (Invitrogen, Carlsbad, CA). Cells were harvested 48 h post‐transfection for further analysis.

### 2.4 Real‐time quantitative polymerase chain reaction (RT‐qPCR)

Total RNA was extracted from tumor cells using TRIzol reagent (Invitrogen). cDNA synthesis was performed using the iScript cDNA Kit (Bio‐Rad, Hercules, CA). RT‐qPCR was carried out with SYBR Premix Ex Taq™ (Takara, Dalian, China) on a CFX96 Touch real‐time PCR system (Bio‐Rad). Relative expression levels of miRNAs and LINC01871/mRNAs were normalized to U6 or GAPDH and quantified using the 2^−ΔΔCt^ method. Primer sequences are provided in Table S1.

### 2.5 Subcellular fractionation assay

Nuclear and cytoplasmic fractions of CC cells were isolated using the Nuclear/Cytosol Fractionation Kit (ab289882, Abcam, Shanghai, China) following the manufacturer's instructions. LINC01871 was extracted from both fractions and quantified by RT‐qPCR, with U6 and GAPDH serving as endogenous controls for the nuclear and cytoplasmic fractions, respectively.

### 2.6 Transwell assay

Tumor cell invasiveness and migration were assessed using 24‐well Transwell chambers (8‐μm pore size; Corning Inc., Corning, NY). For the migration assay, 1 × 10^4^ transfected CC cells in 200 μL of serum‐free medium were seeded into the upper chamber, while the lower chamber contained 500 μL of complete medium with 10% FBS. After 24 h, cells that had migrated to the lower surface of the membrane were fixed with 4% paraformaldehyde and stained with crystal violet for 10 min. For the invasion assay, the upper chamber was precoated with 100 μL of Matrigel (Beyotime, Shanghai, China), and the remaining steps followed those of the migration assay. Cells were imaged using a microscope (Leica Microsystems, Shanghai, China), and five random fields were selected to count the number of migrating and invading cells.

### 2.7 Western blotting

Proteins were extracted from cells or tumor tissues using RIPA buffer (Solarbio, Beijing, China), and their concentrations were quantified using a bicinchoninic acid assay kit (Beyotime). Equal amounts of protein (20 μg) were resolved on 10% SDS‐PAGE gels, transferred onto polyvinylidene fluoride membranes (Beyotime), and blocked with 5% defatted milk in Tris‐buffered saline with 0.1% Tween‐20 (TBS‐T). The membranes were then incubated with primary antibodies (detailed in Table S2) at 4°C overnight, followed by three washes in TBS‐T. Subsequently, the membranes were incubated with horseradish peroxidase‐conjugated secondary antibody (ab7090, Abcam) for 2 h at room temperature. Lastly, protein bands were visualized using an enhanced chemiluminescence detection kit (Solarbio) and quantified with ImageJ software.

### 2.8 In vitro macrophage phagocytosis assay

Human peripheral blood monocytes, obtained from Procell (Wuhan, China), were cultured in RPMI‐1640 medium supplemented with 10% FBS. To induce macrophage differentiation, monocytes were treated with 100 ng/mL recombinant human macrophage‐colony stimulating factor (M‐CSF; Solarbio) for 6 days. The phagocytic activity of the resulting macrophages was assessed by seeding 1 × 10^5^ macrophages into a 24‐well plate and labeling them with fluorescent Dil dye (5 μM; D3911, Invitrogen). Concurrently, CC cells (1 × 10^5^) were labeled with CFDA‐SE fluorescent dye (5 μM; C1031, Beyotime) and cocultured with macrophages in the same well for 4 h. Phagocytic interactions were visualized using a fluorescence microscope (Leica Microsystems) and quantitatively analyzed with a flow cytometer (Beckman Coulter, Brea, CA).

### 2.9 Luciferase reporter assay

The putative binding sites between LINC01871 and miR‐873‐3p, as well as between miR‐873‐3p and MAP3K2, were predicted using the miRDB database (https://mirdb.org/). Mutations in the predicted binding sequences of LINC01871 or the MAP3K2 3′ untranslated region (3′UTR) were introduced using the QuickMutation™ Site‐Directed Mutagenesis Kit (Beyotime). Wild‐type (Wt) and mutant (Mut) sequences of LINC01871 or MAP3K2 3′UTR were subcloned into the pmirGLO vector (Promega, Madison, WI) to generate pmirGLO‐LINC01871‐Wt/Mut and pmirGLO‐MAP3K2 3′UTR‐Wt/Mut constructs. These plasmids were co‐transfected with miR‐873‐3p mimic or miR‐NC into CC cells. After 48 h, luciferase activity was measured using the Dual‐Luciferase Reporter Assay System (Promega).

### 2.10 Xenograft mouse model

Ten 4‐week‐old female BALB/c nude mice were purchased from Vital River (Beijing, China) and maintained under specific pathogen‐free conditions. Following a one‐week acclimatization period, the mice were randomly assigned to two groups: the sh‐NC group and the sh‐LINC01871 group (*n* = 5/group). HeLa cells (2 × 10^6^) transfected with either sh‐NC or sh‐LINC01871, were subcutaneously injected into the right flanks of the mice. Tumor volume was measured every 4 days using the formula: tumor volume (mm^3^) = width^2^ × length × 0.5. On day 28, all mice were euthanized under anesthesia via cervical dislocation, and the tumors were harvested for subsequent analyses. All animal experiments were performed following the guidelines and approved by the Ethics Committee of the First Affiliated Hospital of Bengbu Medical University (Ethical Animal Science No. 351 [2024]).

### 2.11 Statistical analysis

Data are presented as mean ± standard deviation. Each experiment was performed in triplicate or more. Statistical differences between two groups were evaluated using Student's *t*‐test, while comparisons among multiple groups were conducted using one‐way ANOVA followed by Tukey's post hoc analysis. Analyses were performed using GraphPad Prism 8.0.2 (GraphPad, San Diego, CA). A *p* <0.05 indicated statistical significance.

## 3 RESULTS

### 3.1 LINC01871 silencing suppresses CC cell metastasis and EMT

**FIGURE 1:**
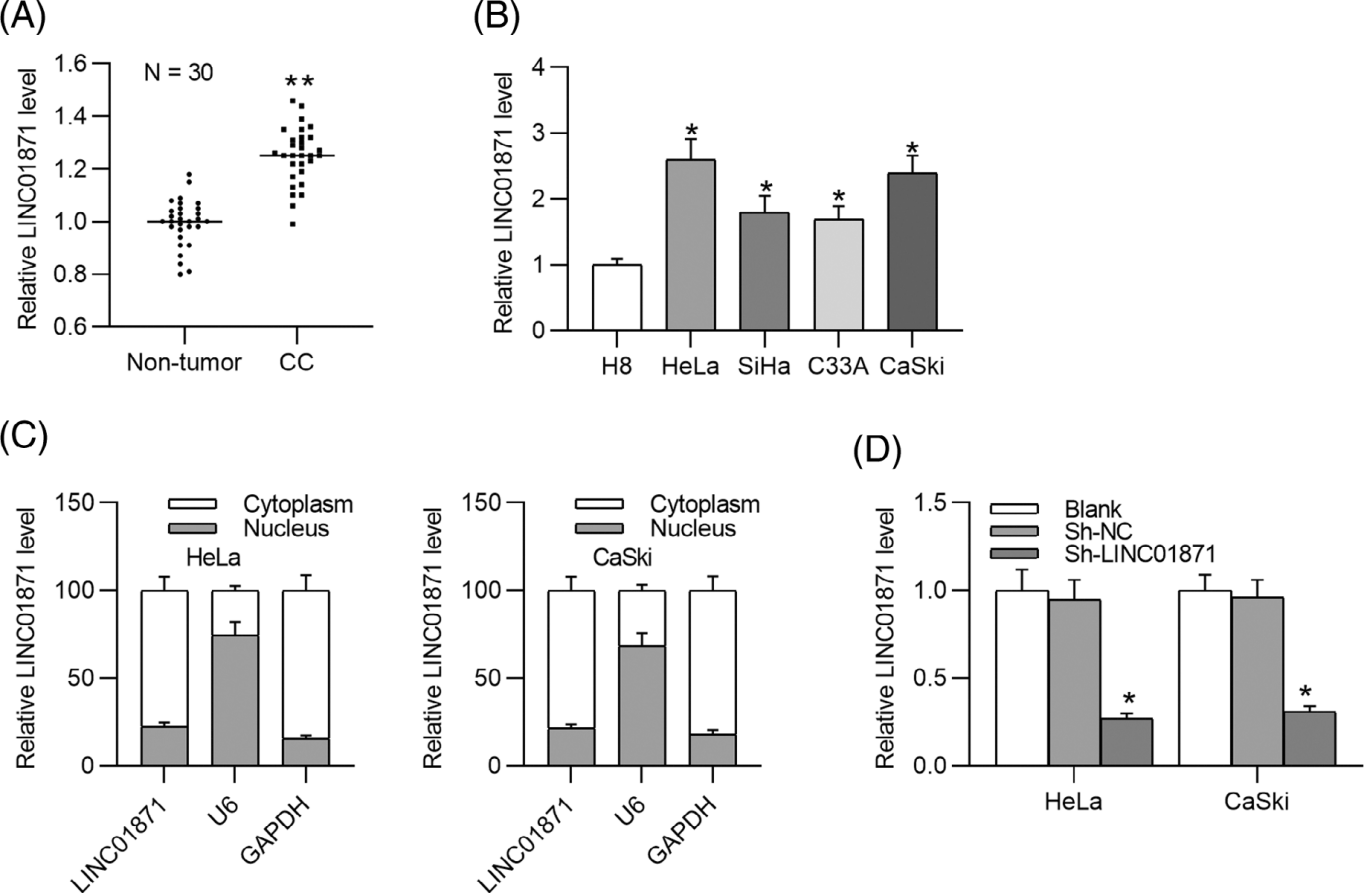
Expression and subcellular localization of LINC01871 in cervical cancer (CC) cells. (A) Real‐time quantitative polymerase chain reaction (RT‐qPCR) analysis of LINC01871 expression in CC samples and adjacent nontumor tissues (*N* = 30). (B) RT‐qPCR analysis of LINC01871 expression in the normal cervical epithelial cell line (H8) and CC cell lines (HeLa, CaSki, C33A, SiHa). (C) Subcellular fractionation assay to determine the distribution of LINC01871 in CC cells. (D) RT‐qPCR analysis to confirm the knockdown efficiency of LINC01871 in CC cells transfected with sh‐LINC01871 or sh‐NC. *p <0.05; **p <0.01.

**FIGURE 2:**
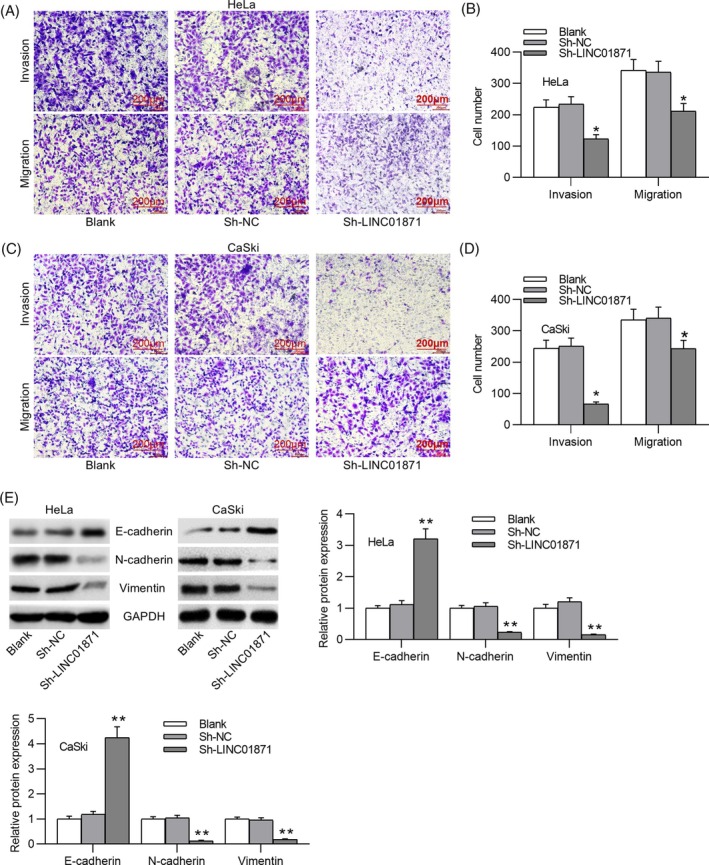
LINC01871 silencing restrains cervical cancer (CC) cell metastasis and EMT. (A–D) Transwell assays for evaluating the invasion and migration of HeLa (A, B) and CaSki cells (C, D). (E) Western blotting for assessing epithelial–mesenchymal transition (EMT)‐related protein levels in the indicated CC cell lines. *p <0.05; **p <0.01.

We first analyzed LINC01871 expression in patient‐derived tissues. RT‐qPCR results demonstrated significantly higher LINC01871 expression in CC tissues compared to adjacent nontumor tissues (Figure 1A). Similarly, LINC01871 expression was markedly elevated in CC cell lines compared to the normal cervical epithelial H8 cell line (Figure 1B). Among the CC cell lines examined, HeLa and CaSki, which exhibited relatively higher LINC01871 expression, were selected for subsequent experiments. Subcellular fractionation assays revealed that LINC01871 is predominantly localized in the cytoplasm of HeLa and CaSki cells, suggesting a potential role in post‐transcriptional regulation (Figure 1C). To investigate its functional role in CC, we silenced LINC01871 in HeLa and CaSki cells. As depicted in Figure 1D, transfection with sh‐LINC01871 significantly reduced LINC01871 expression levels. Transwell assays depicted that LINC01871 knockdown markedly suppressed the invasion and migration of CC cells (Figure 2A–D). Likewise, silencing LINC01871 enhanced E‐cadherin expression while downregulating Vimentin and N‐cadherin in CC cells (Figure 2E), suggesting that LINC01871 silencing inhibits EMT in CC cells.

### 3.2 LINC01871 depletion enhances macrophage‐mediated phagocytosis and impedes immune escape

**FIGURE 3:**
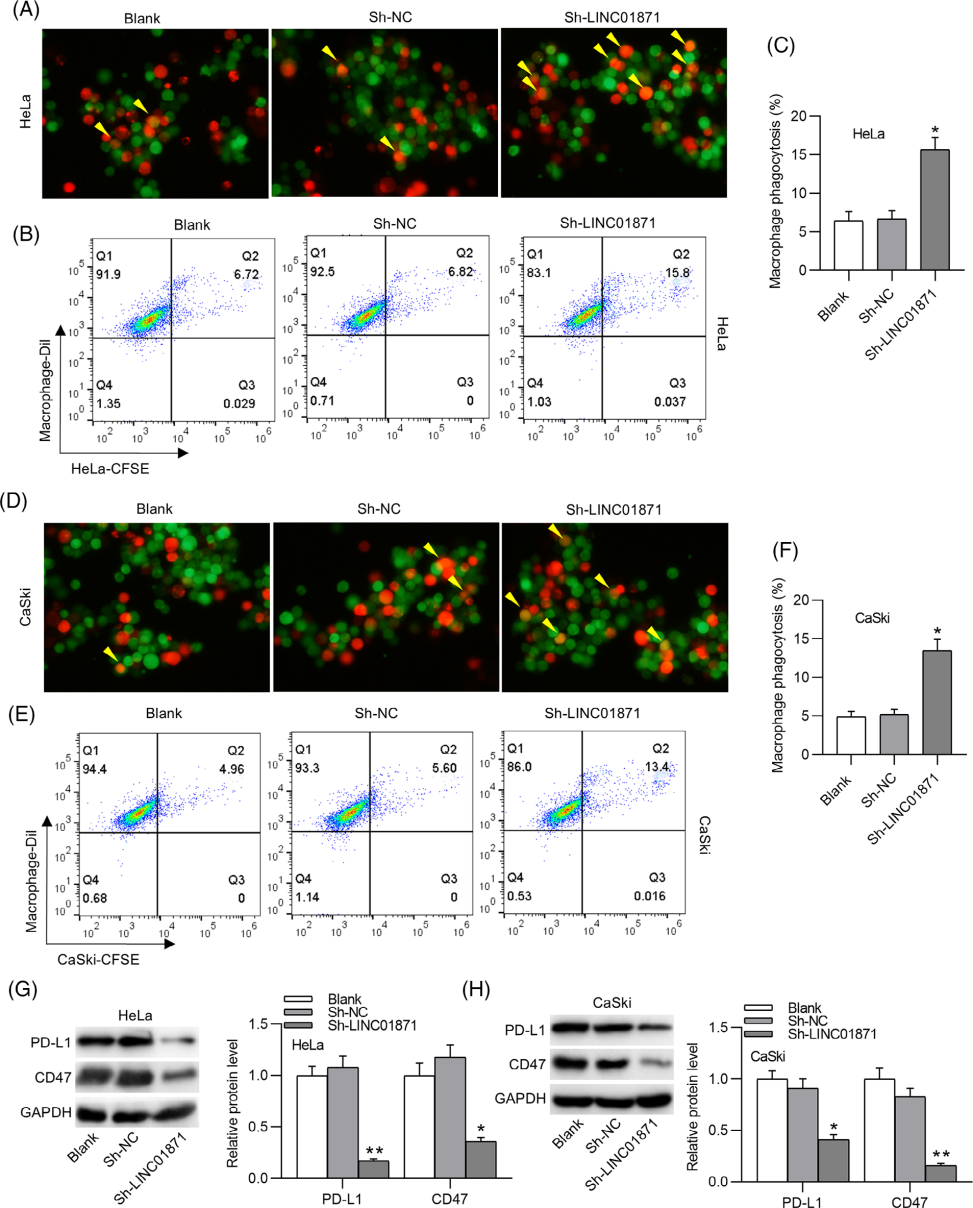
LINC01871 depletion impedes immune escape from macrophage phagocytosis. (A, D) Fluorescence microscopy images showing macrophage phagocytosis in each group (red: macrophages, green: tumor cells). (B, C, E, F) Flow cytometry analysis for detecting macrophage phagocytosis in the indicated groups. (G, H) Western blotting for estimating PD‐L1 and CD47 protein levels in HeLa and CaSki cells. *p <0.05; **p <0.01.

An in vitro phagocytosis assay was conducted to evaluate the impact of LINC01871 on macrophage phagocytic activity. Strikingly, compared to the sh‐NC group, macrophage‐mediated phagocytosis was significantly enhanced in the LINC01871‐depleted group, as evidenced by microscopy (Figure 3A,D) and flow cytometry analyses (Figure 3B,C,E,F). Tumor cells often evade immune surveillance by overexpressing immune checkpoint proteins, such as PD‐L1 and CD47, which inhibit immune cell activity.^15^ Western blotting demonstrated that LINC01871 silencing markedly reduced PD‐L1 and CD47 protein levels in both HeLa and CaSki cells (Figure 3G,H). Collectively, these results suggest that LINC01871 depletion disrupts immune escape mechanisms and enhances antitumor immune responses in CC cells.

### 3.3 LINC01871 interacts with miR‐873‐3p

**FIGURE 4:**
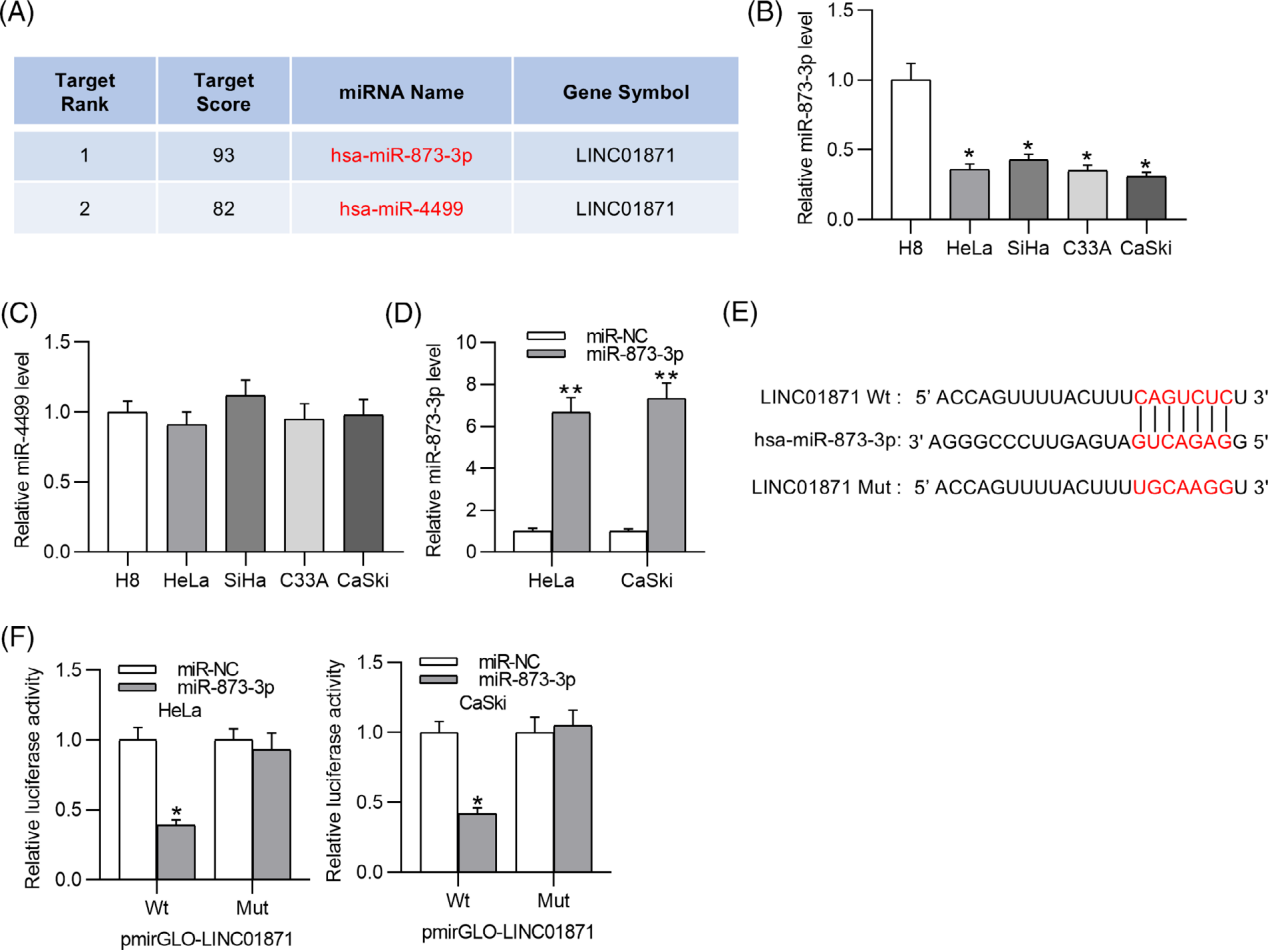
LINC01871 interacts with miR‐873‐3p. (A) Prediction of downstream miRNAs of LINC01871 using the miRDB database (screening criteria: target score >80). (B, C) Real‐time quantitative polymerase chain reaction (RT‐qPCR) of miR‐873‐3p (B) and miR‐4499 (C) expression in the normal cell line and cervical cancer (CC) cell lines. (D) RT‐qPCR for determining miR‐873‐3p overexpression efficiency in CC cells. (E) The putative binding site between miR‐873‐3p and LINC01871 predicted from the miRDB database. (F) Luciferase reporter assay for elucidating the binding ability between miR‐873‐3p and LINC01871. *p <0.05; **p <0.01.

To investigate the underlying mechanism by which LINC01871 regulates CC cell behavior, the miRDB database was utilized to predict its potential downstream miRNAs. Using a target score threshold of >80, two candidate miRNAs (miR‐873‐3p, miR‐4499) were identified (Figure 4A). Expression analysis revealed that miR‐873‐3p was downregulated in CC cell lines (Figure 4B), while no significant difference in miR‐4499 expression was observed across different cell lines (Figure 4C). Therefore, miR‐873‐3p was selected for further investigation. To confirm the interaction between miR‐873‐3p and LINC01871, we overexpressed miR‐873‐3p in CC cells (Figure 4D) and introduced mutations in the predicted binding site on LINC01871 (Figure 4E). Luciferase reporter assays demonstrated that miR‐873‐3p overexpression prominently weakened the luciferase activity of LINC01871‐Wt while having no discernible effect on LINC01871‐Mut (Figure 4F). These results validate the direct binding relationship between LINC01871 and miR‐873‐3p in CC cells.

### 3.4 LINC01871 competitively interacts with miR‐875‐3p to upregulate MAP3K2 and activate MAPK signaling

**FIGURE 5:**
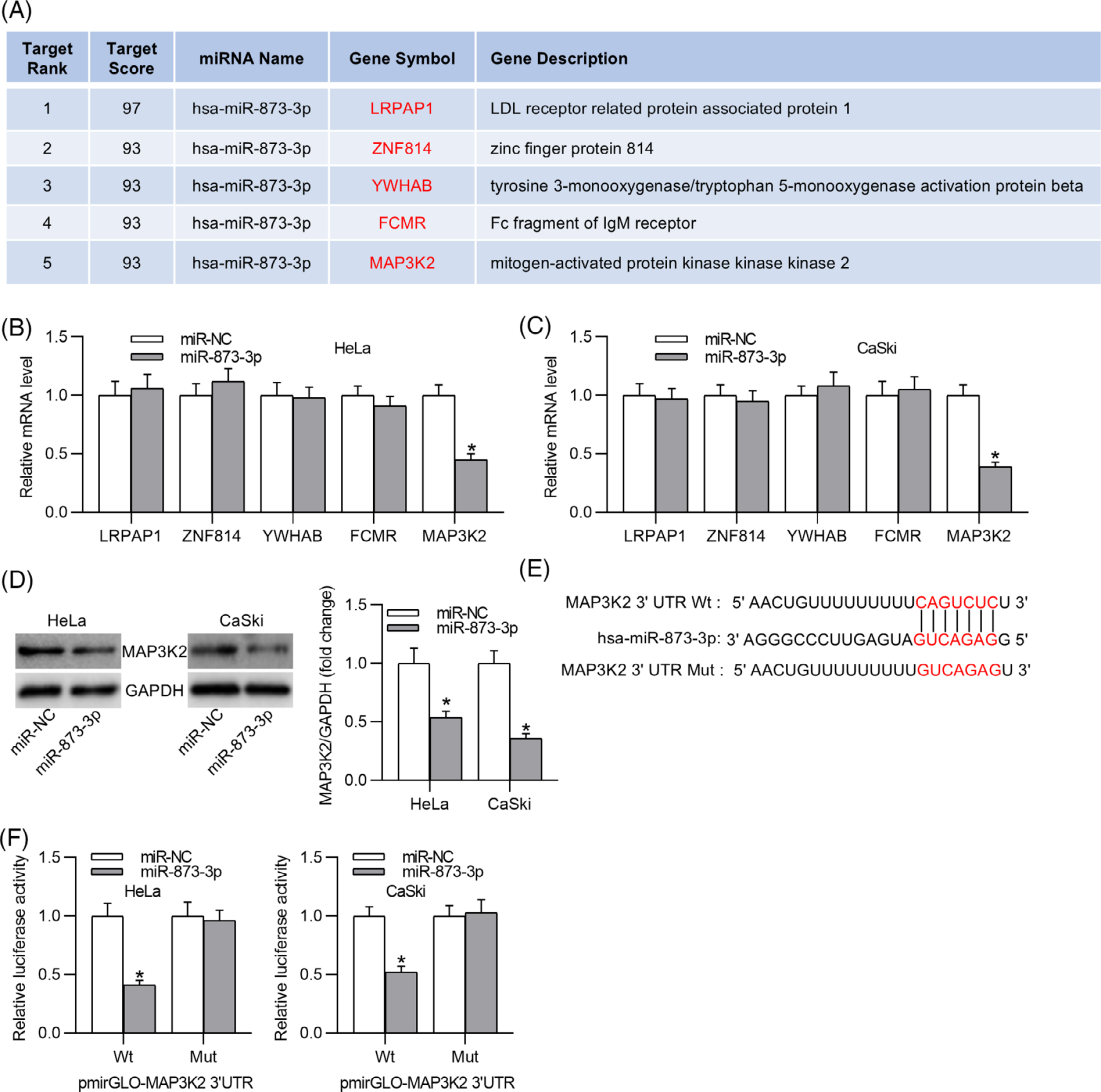
miR‐875‐3p targets MAP3K2. (A) Prediction of potential target genes of miR‐873‐3p using miRDB database (screening condition: target score >92). (B, C) Real‐time quantitative polymerase chain reaction for detecting mRNA levels of the predicted candidates in miR‐873‐3p‐overexpressed HeLa and CaSki cells. (D) Western blotting depicting MAP3K2 protein expression in miR‐973‐3p‐upregulated cervical cancer cells. (E) The putative binding site between MAP3K2 3′UTR and miR‐873‐3p from the miRDB database. (F) Luciferase reporter assay verifying the binding relation between the two molecules. *p <0.05.

**FIGURE 6:**
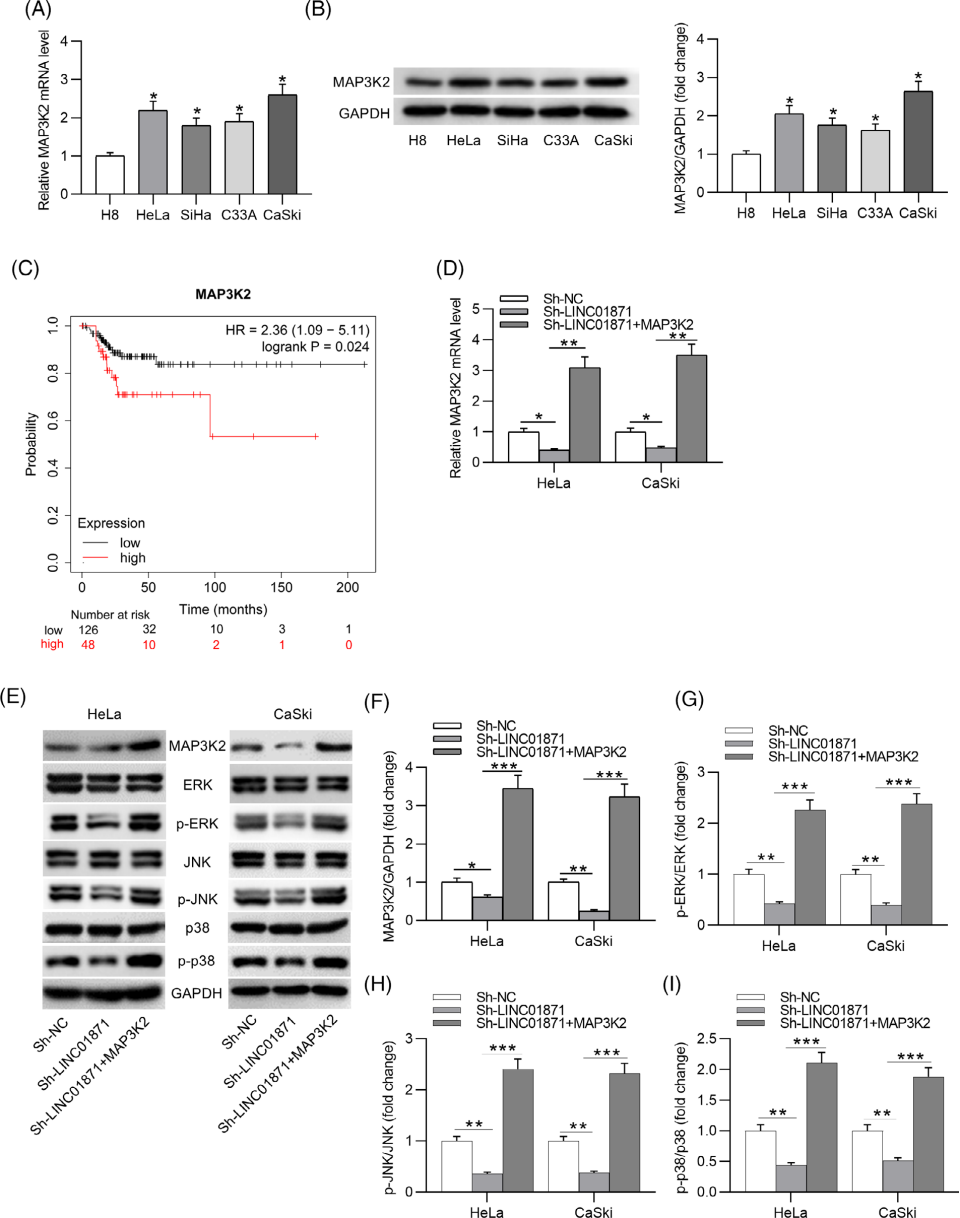
MAP3K2 is upregulated in cervical cancer (CC) cells. (A, B) Real‐time quantitative polymerase chain reaction (RT‐qPCR) and western blotting for detecting MAP3K2 mRNA (A) and protein (B) expression in CC and normal H8 cell lines. (C) GEPIA database analysis shows the correlation between MAP3K2 expression and CC patient overall survival rate. (D) RT‐qPCR of MAP3K2 mRNA level in CC cells with different treatments. (E) Representative western blot images showing protein levels of MAP3K2 and MAPK signaling‐related markers. (F–I) Quantitative results of western blotting. *p <0.05; **p <0.01; ***p <0.001.

We next investigated the target genes of miR‐873‐3p using the miRDB database. Five candidate genes were identified based on a target score threshold of >92 (Figure 5A). Among these, only MAP3K2 expression was significantly downregulated in CC cells overexpressing miR‐873‐3p (Figure 5B,C). Western blotting further confirmed a marked reduction in MAP3K2 protein levels in miR‐873‐3p‐overexpressing CC cells (Figure 5D). The miRDB database predicted a binding site for miR‐873‐3p on MAP3K2 (Figure 5E), which was validated through a luciferase reporter assay (Figure 5F). Furthermore, both MAP3K2 mRNA and protein levels were significantly elevated in CC cell lines compared to the H8 cell line (Figure 6A,B). Data retrieved from the GEPIA database further demonstrated that CC patients with high MAP3K2 expression exhibited poorer prognoses compared to those with low expression (Figure 6C). Silencing LINC01871 in CC cells resulted in reduced MAP3K2 expression; this effect was reversed upon overexpression of MAP3K2 (Figure 6D–F). To elucidate the mechanism underlying the LINC01871/miR‐183‐3p/MAP3K2 axis in promoting the malignant behavior of CC cells, we examined its impact on the MAPK signaling pathway, which is critically implicated in tumorigenesis. Western blotting revealed that LINC01871 knockdown decreased the phosphorylation levels of signal‐regulated kinase (ERK), c‐Jun N‐terminal kinase (JNK), and p38 in CC cells. In contrast, MAP3K2 overexpression restored the protein levels of p‐ERK, p‐JNK, and p‐p38 (Figure 6E,G–I). Taken together, these findings demonstrate that LINC01871 promotes MAP3K2 expression and activates the MAPK signaling pathway by competitively binding to miR‐873‐3p in CC cells.

### 3.5 Overexpression of MAP3K2 reverses LINC01871 silencing‐mediated effects on CC cell metastasis and immune evasion

**FIGURE 7:**
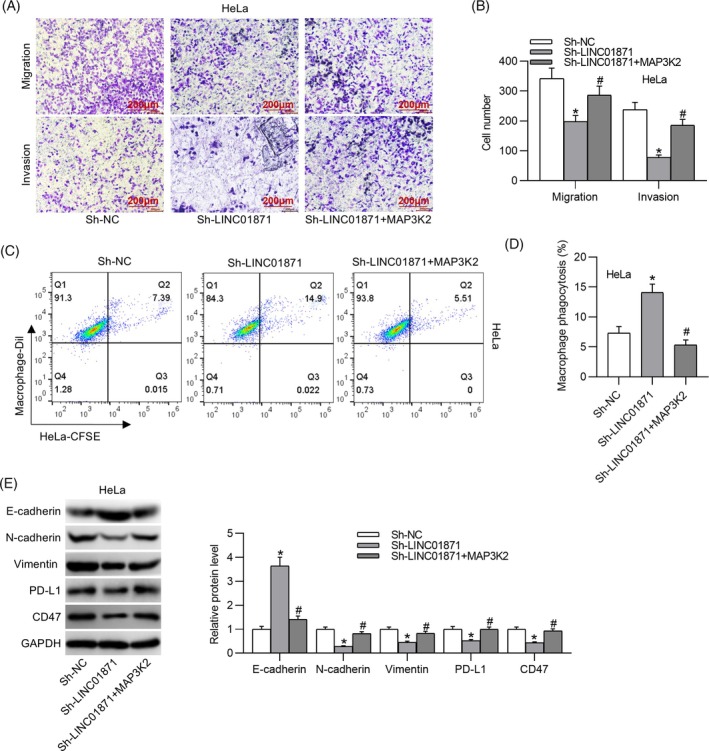
LINC01871 promotes cervical cancer cell metastasis and immune escape by upregulating MAP3K2. (A, B). Transwell assays for assessing HeLa cell invasion and migration. (C, D) Flow cytometry for evaluating macrophage phagocytosis. (E) Western blotting for determining protein levels of epithelial–mesenchymal transition‐related markers, PD‐L1, and CD47 in the indicated HeLa cells. *p <0.05 versus sh‐NC; #*p* <0.05 versus sh‐LINC01871.

Rescue experiments were performed to substantiate whether LINC01871 modulates CC cell behaviors through MAP3K2 upregulation. Notably, MAP3K2 overexpression effectively reversed the suppression of CC cell invasion and migration caused by LINC01871 silencing (Figure 7A,B). Furthermore, the enhanced macrophage phagocytosis observed following LINC01871 depletion was significantly attenuated by MAP3K2 overexpression, as evidenced by flow cytometry (Figure 7C,D). Similarly, MAP3K2 overexpression counteracted the LINC01871 knockdown‐induced upregulation of E‐cadherin, along with the downregulation of N‐cadherin, Vimentin, PD‐L1, and CD47 in HeLa cells (Figure 7E). These findings suggest that LINC01871 facilitates EMT and immune evasion in CC cells by modulating MAP3K2 expression.

### 3.6 Silencing LINC01871 suppresses tumor growth in a xenograft mouse model

**FIGURE 8:**
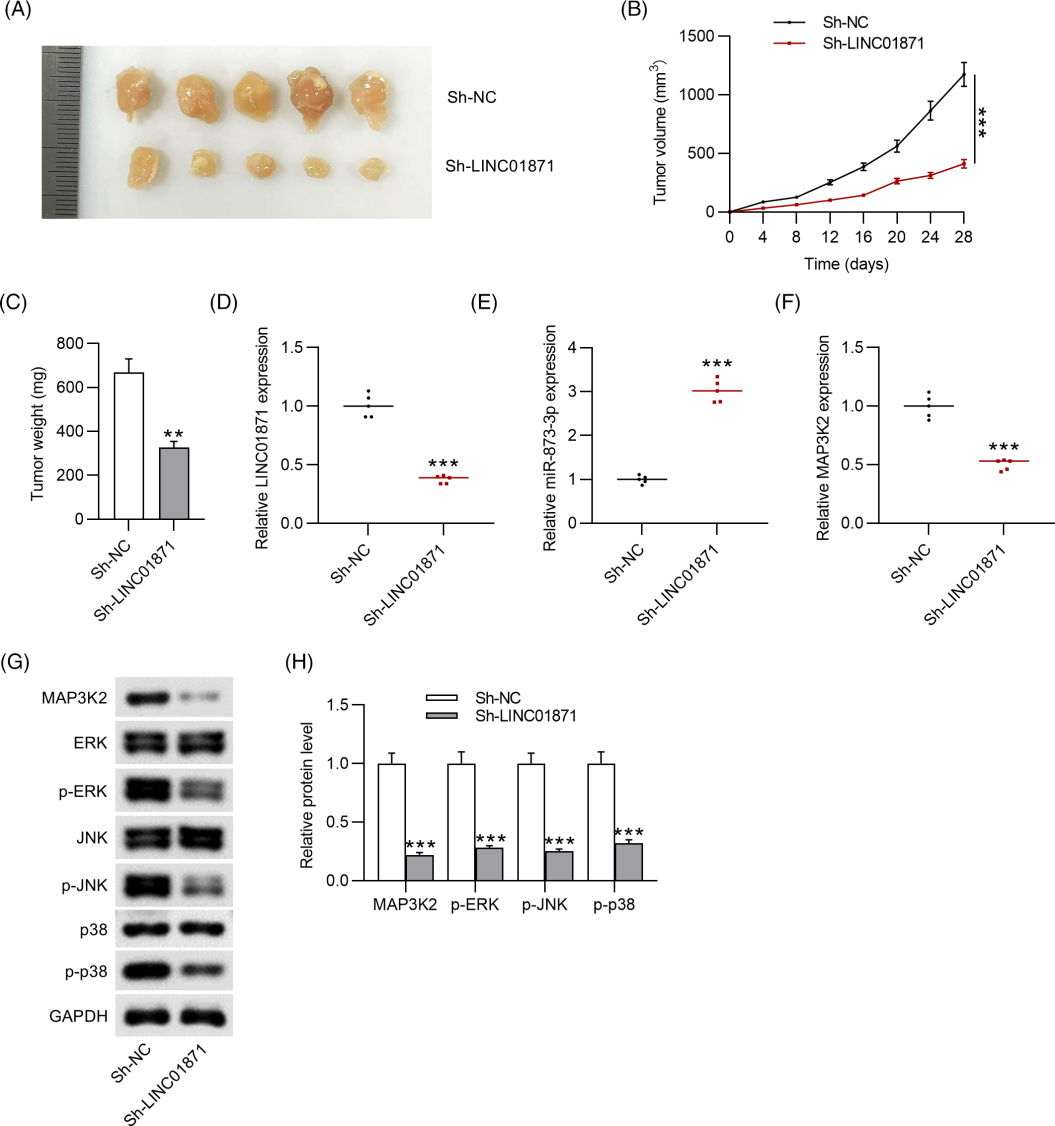
LINC01871 silencing inhibits tumor growth of CC in vivo. (A) Tumor images from sh‐NC and sh‐LINC01871 groups. (B) Tumor volume was monitored every 4 days. (C) Tumor weight on the 28th day. (D–F) Real‐time quantitative polymerase chain reaction analysis of LINC01871, miR‐183‐3p, and MAP3K2 expression in tumors of the sh‐NC and sh‐LINC01871 groups. (G) Representative images of western blotting showing protein levels of MAP3K2 and MAPK signaling‐related markers in tumor tissues. (H) Quantitative results of western blotting. **p <0.01; ***p <0.001.

To further elucidate the carcinogenic role of LINC01871 in CC, a xenograft mouse model was established by injecting HeLa cells transduced with sh‐LINC01871 into nude mice. Tumors in the sh‐LINC01871 group were significantly smaller in size and lighter in weight compared to those in the sh‐NC group (Figure 8A–C), demonstrating that LINC01871 knockdown inhibited HeLa cell‐derived tumor growth in vivo. Moreover, LICN01871 silencing suppressed the expression of LINC01871 and MAP3K2 while upregulating miR‐873‐3p levels in tumor tissues (Figure 8D–F). Consistent with the in vitro findings, animal experiments showed that LINC01871 knockdown downregulated MAP3K2 protein expression and suppressed the phosphorylation of ERK, JNK, and p38 in tumor‐bearing mice (Figure 8G,H). These results highlight the promotive effect of LINC01871 in the tumorigenesis of CC in vivo.

## 4 DISCUSSION

Accumulating evidence has highlighted the pivotal role of lncRNAs as critical regulators in tumor initiation and progression.^16^ It has been reported that overexpression of LINC01871 inhibits CC cell viability and enhances cell sensitivity to anticancer agents through the miR‐142‐3p/ZYG11B axis.^17^ To our knowledge, this study is the first to reveal the effect of LINC01871 on CC cell metastasis.

EMT, characterized by the loss of epithelial markers such as E‐cadherin and the acquisition of mesenchymal markers like Vimentin and N‐cadherin, plays a critical role in promoting tumor cell invasion and metastasis.^18^ Several lncRNAs, such as HOTAIR and PTENP1, have been shown to promote EMT in CC, thereby contributing to its progression.^19^, ^20^ In our study, silencing LINC01871 significantly suppressed CC cell invasiveness, migration, and EMT. Furthermore, tumor cells often overexpress immune checkpoint molecules such as CD47 and PD‐L1 to evade immune system attack, facilitating their survival and metastasis to lymph nodes.^21^ CD47 interacts with its receptor, signal‐regulated protein alpha (SIRPα) on macrophages, inhibiting phagocytosis and contributing to immune evasion.^22^ Cytotoxic T cells, key effectors of the adaptive immune response, form the cornerstone of cancer immunotherapy.^23^ However, PD‐L1 binding to its receptor PD‐1 on activated T cells impedes antitumor immunity by disrupting T cell activation signals, a critical mechanism enabling tumor cells to evade adaptive immune surveillance.^24^ PD‐L1/PD‐1 blocking antibodies have been employed in cancer immunotherapy to reinvigorate exhausted T cells and restore pre‐existing antitumor immunity.^25^ Additionally, studies suggest that macrophages within the tumor microenvironment also express PD‐1, and blocking PD‐1/PD‐L1 interaction enhances macrophage phagocytosis in the mouse model.^26^ In our study, LINC01871 was found to be upregulated in CC tissue samples, and silencing of LINC01871 prominently elevated macrophage phagocytosis and reduced PD‐L1 and CD47 expression in CC cells, indicating that LINC01871 retrained CC cell metastasis by impeding immune escape. Furthermore, animal experiments revealed that LINC01871 silencing inhibited tumor growth derived from HeLa cells in a xenograft mouse model, further confirming the oncogenic role of LINC01871 in CC.

Evidence demonstrates that lncRNAs exhibit distinct functions based on their subcellular localization.^27^ Our findings confirm the cytoplasmic localization of LINC01871 in CC cells. It is well‐established that in the cytoplasm, lncRNAs can indirectly modulate the stability and translation of mRNAs by interacting with shared miRNAs.^28^ As previously mentioned, LINC01871 upregulates ZYG11B expression by sponging miR‐142‐3p in CC cells,^17^ indicating the role of LINC01871 at post‐transcription. Similarly, our results demonstrated that LINC01871 interacted with miR‐873‐3p to enhance MAP3K2 expression. Previous reports have revealed the involvement of miR‐873‐3p in various cancers. Importantly, a recent report proposed that lncRNA LMCD1‐AS1 enhances CC cell proliferation and EMT by binding to miR‐873‐3p,^29^ which is consistent with our findings that miR‐873‐3p was downregulated in CC cell lines.

MAP3K2, also known as MEKK2, is a member of the serine/threonine protein kinase family and plays a crucial role in regulating the MAPK signaling pathway.^30^ Studies have shown that MAP3K2 is implicated in the progression of various malignancies. For example, MAP3K2 overexpression has been shown to reverse the miR‐335‐mediated inhibition of breast cancer cell proliferation.^31^ In colon cancer, miR‐372‐3p targets MAP3K2, thereby suppressing tumor development.^32^ These findings underscore the oncogenic potential of MAP3K2 in cancer. However, its role as an oncogene in CC remains unclear. Consistent with the aforementioned studies, our data show that MAP3K2 is upregulated in CC cell lines, and its overexpression significantly counteracted the inhibitory effects of LINC01871 silencing on EMT and immune escape in CC cells. The MAPK signaling pathway, which involves three key components (ERK, JNK, p38), is a pivotal regulator of numerous cellular processes, including cell proliferation, apoptosis, and migration.^33^ Targeting this pathway has emerged as a promising strategy for cancer treatment.^34^ Our results indicated that MAP3K2 overexpression reversed the suppression of MAPK pathway activation induced by LINC01871 silencing in CC cells, suggesting that the activation of the MAPK signaling cascade is central to the LINC01871/miR‐183‐3p/MAP3K2 axis‐mediated enhancement of CC cell aggressiveness.

In conclusion, this study demonstrates that LINC01871 promotes CC cell metastasis by enhancing EMT and facilitating immune evasion from macrophage phagocytosis through the miR‐873‐3p/MAP3K2/MAPK signaling pathway. These findings provide valuable insights that could inform the development of novel therapeutic strategies for CC.

## CONFLICT OF INTEREST STATEMENT

The authors declare no competing interests.

## Supporting information


**Table S1.** Primer sequences used in RT‐qPCR.
**Table S2.** Primary antibodies used in western blotting.

## Data Availability

The data that support the findings of this study are available from the corresponding author upon reasonable request.
