# Supplementary material for: LINC01871 facilitates cervical cancer cell migration and immune escape by targeting miR‐873‐3p/MAP3K2 axis
Source: Kaohsiung J Med Sci. 2025 Mar 4;41(4):e12948. doi: 10.1002/kjm2.12948 (PMC11964103; doi:10.1002/kjm2.12948)
Supplement: Supplementary file 1 — Table S1. Primer sequences used in RT‐qPCR. Table S2. Primary antibodies used in western blotting. [file KJM2-41-e12948-s001.docx]

**Table S1. Primer sequences used in RT-qPCR.**

| Gene | Forward (5’-3’) | Reverse (5’-3’) |
| --- | --- | --- |
| LINC01871 | TCTCCCTATTCCTTACTTG | AGCCCACTGATAATGTCT |
| miR-873-3p | TTTGTGTGCATTTGCAGGAACT | GAAGATTTGTGGGTGTTCCCG |
| miR-4499 | GCGAGGGCCCTTGAGTAGT | AGTGCAGGGTCCGAGGTATT |
| LRPAP1 | GAGTTCCTGCATCACAAAGAG | CTCGTGGATTTCTTCGGTC |
| ZNF814 | TACTCAGTCAGCACCAGAG | GCATATTTGCTAAAGGACTTCC |
| YWHAB | CAAACAAACCACTGTGTCGA | CCAAGACGAATTGGGTGTG |
| FCMR | TACTTCCTGCCAGTATCGG | TGATGGTAACTGATCCGCC |
| MAP3K2 | CAGTCGACCAGCATTATCC | TTGACTCGGACATCATTCTG |
| U6 | ATACAGAGAAAGTTAGCACGG | GGAATGCTTCAAAGAGTTGTG |
| GAPDH | TCAAGATCATCAGCAATGCC | CGATACCAAAGTTGTCATGGA |

**Table S2. Primary antibodies used in western blotting.**

| Target | Host species/Clonality | Cat No.* | Concentration |
| --- | --- | --- | --- |
| E-cadherin | Rabbit monoclonal | ab40772 | 1:1000 |
| N-cadherin | Rabbit monoclonal | ab76011 | 1:5000 |
| Vimentin | Rabbit monoclonal | ab92547 | 1:1000 |
| PD-L1 | Rabbit monoclonal | ab205921 | 1:1000 |
| CD47 | Rabbit monoclonal | ab300124 | 1:1000 |
| MAP3K2 | Rabbit monoclonal | ab240926 | 1:10000 |
| ERK | Rabbit monoclonal | ab184699 | 1:10000 |
| p-ERK | Rabbit monoclonal | ab201015 | 1:1000 |
| JNK | Rabbit monoclonal | ab179461 | 1:1000 |
| p-JNK | Rabbit monoclonal | ab76572 | 1:5000 |
| p38 | Rabbit monoclonal | ab170099 | 1:1000 |
| p-p38 | Rabbit monoclonal | ab195049 | 1:1000 |
| GAPDH | Rabbit polyclonal | ab9485 | 1:2500 |
| *From Abcam, Shanghai, China | | | |
